# *FBP1 /*miR-24-1/enhancer axis activation blocks renal cell carcinoma progression *via* Warburg effect

**DOI:** 10.3389/fonc.2022.928373

**Published:** 2022-08-01

**Authors:** Dongen Ju, Ying Liang, Guangdong Hou, Wanxiang Zheng, Geng Zhang, Xinlong Dun, Di Wei, Fei Yan, Lei Zhang, Dong Lai, Jiarui Yuan, Yu Zheng, Fuli Wang, Ping Meng, Yong Wang, Wenqiang Yu, Jianlin Yuan

**Affiliations:** ^1^ Department of Urology, Xijing Hospital, Fourth Military Medical University, Xi’an, China; ^2^ Laboratory of RNA Epigenetics, Institutes of Biomedical Sciences, Shanghai Medical College, Fudan University, Shanghai, China; ^3^ Shanghai Public Health Clinical Center and Department of General Surgery, Huashan Hospital, Cancer Metastasis Institute, Fudan University, Shanghai, China; ^4^ Department of Pharmacy, Precision Pharmacy and Drug Development Center, Tangdu Hospital, Fourth Military Medical University, Xi’an, China; ^5^ Clinical Medicine Department, St. George’s University School of Medicine, Saint George, Grenada; ^6^ Medical Innovation Center, Fourth Military Medical Univeristy, Xi’an, China; ^7^ Department of Urology, Tangdu Hospital, Fourth Military Medical University, Xi’an, China

**Keywords:** *FBP1*, renal cell carcinoma, enhancer, Warburg Effect, miR-24-1

## Abstract

Warburg effect is a pivotal hallmark of cancers and appears prevalently in renal cell carcinoma (RCC). FBP1 plays a negative role in Warburg effect as a rate-limiting enzyme in gluconeogenesis, yet its mechanism in RCC remains to be further characterized. Herein, we revealed that *FBP1* was downregulated in RCC tissue samples and was related to the poor survival rate of RCC. Strikingly, miR-24-1 whose DNA locus is overlapped with enhancer region chr9:95084940-95087024 was closely linked with the depletion of *FBP1* in RCC. Of note, miRNAs like miR-24-1 whose DNA loci are enriched with H3K27ac and H3K4me1 modifications are belonging to nuclear activating miRNAs (NamiRNAs), which surprisingly upregulate target genes in RCC through enhancer beyond the conventional role of repressing target gene expression. Moreover, miR-24-1 reactivated the expression of *FBP1* to suppress Warburg effect in RCC cells, and subsequently inhibited proliferation and metastasis of RCC cells. In mechanism, the activating role of miR-24-1 was dependent on enhancer integrity by dual luciferase reporter assay and CRISPR/Cas9 system. Ultimately, animal assay *in vivo* validated the suppressive function of *FBP1* on 786-O and ACHN cells. Collectively, the current study highlighted that activation of *FBP1* by enhancer-overlapped miR-24-1 is capable of contributing to Warburg effect repression through which RCC progression is robustly blocked, providing an alternative mechanism for RCC development and as well implying a potential clue for RCC treatment strategy.

## Introduction

Renal cell carcinoma (RCC) is the most common type of kidney cancer with increasing incidence and mortality rates (1). The main subtypes of RCC include clear cell renal cell carcinoma (ccRCC), papillary renal cell carcinoma (pRCC) and chromophobe RCC (ChRCC), which account for 65–70%, 15–20%, and 5–7% of total RCC cases, respectively (2). It was highlighted that inactivation of the *VHL* tumor suppressor gene caused by biallelic mutation or promoter hypermethylation is involved in the majority of RCCs with characteristic metabolic alterations (3, 4). However, kidney-specific *VHL* deletion in mice is not sufficient to induce RCC-specific metabolic changes or tumorigenesis, indicating that alternative mechanisms are concealed (5).

Altered energy metabolism is widely recognized as a hallmark of cancer and stretches beyond adaptations to support the increased energy requirements of unrestrictedly growing and dividing cancer cells (1, 6). Typically, cancer cells take up increased amounts of glucose to produce elevated levels of the glycolytic metabolite pyruvate compared to normal cells, which is preferentially converted to lactate by lactate dehydrogenase (LDH) even in the presence of oxygen, yielding a large amount of ATP in a short-circuit pattern (7). This type of metabolism is taken as Warburg effect, characterized by drastically increased glycolytic rates and lactate production (1, 8). Of note, FBP1 plays a negative role in Warburg effect by catalyzing the hydrolysis process of fructose 1,6-bisphosphate to fructose 6-phosphate (9, 10). Given that *FBP1* is downregulated in RCC as a tumor suppressor and that its depletion enhances HIF activity to suppress kidney cancer (11), we want to investigate the underlying molecular mechanism of Warburg effect in RCC through *FBP1*.

Notably, accumulating evidence indicates that dysfunction of *FBP1* can be regulated by ectopic expression of miRNAs (9, 10, 12). To our knowledge, miRNAs post transcriptionally degrade or repress target genes *via* binding to the 3’ UTR of their mRNAs, yet relatively little is known about their regulatory role in transcriptional activation. In our previous work (13), we revealed a type of miRNA whose DNA loci are overlapped with enhancer region can activate the expression of target genes through the corresponding enhancers (named NamiRNA), in particular, *FBP1* was activated by the enhancer-overlapping NamiRNA-24-1 in a manner dependent on enhancer activity in HEK293T cells. Thus, we proposed a NamiRNA-enhancer-gene activation network to better understand the miRNA activation phenomenon (14–16). Other findings also support the critical crosstalk between enhancers and their overlapping miRNAs, highlighting important tissue-specific cancer biomarkers (17, 18). Moreover, low expression of miRNAs is another feature of cancer cell. It is reported miR-24-1 can function as a tumor-suppressive miRNA in cancer development (19, 20). Therefore, we wonder whether NamiRNA-24-1 can play activating role on *FBP1* during RCC development.

Herein, we detected by bioinformatic analysis that both *FBP1* and miR-24-1 were downregulated in RCC tissue samples from the TCGA database and further verified their low expression levels in RCC tissue samples by qPCR. Furthermore, through cell-based biological assays, we clarified that overexpression of miR-24-1 can reactivate the expression level of *FBP1* in the ccRCC cell line 786-O and pRCC cell line ACHN, and thus inhibit RCC cell proliferation and migration. Moreover, we confirmed by a dual luciferase reporter assay that the enhancer region containing the miR-24-1 DNA locus can increase reporter gene activity and that its own activity can be enhanced by miR-24-1. Furthermore, we demonstrated that reactivation of *FBP1* by miR-24-1 can inhibit aerobic glycolysis in 786-O and ACHN cells and downregulate the expression of metabolism-related genes involved in the Warburg effect. Finally, overexpression of miR-24-1 suppressed tumor growth of RCC in an animal xenograft model, yet enhancer depletion led to loss of function of miR-24-1, suggesting that reactivation of *FBP1* by miR-24-1 relies on enhancer integrity and can provide a potential treatment strategy for RCC.

## Materials and methods

### Cell culture, and antibodies

The human kidney cancer cell lines 786-O and ACHN and the human embryonic kidney cell line HEK293T were routinely tested to confirm that they were mycoplasma-free. Cell lines were maintained in DMEM (HyClone) at 37°C with 5% CO2. Cultures were coated with 10% fetal bovine serum (FBS, HyClone) and 1% penicillin/streptomycin (HyClone). All cells used were expanded less than 6 months after resuscitation. The primary antibodies were anti-H3K27ac (ab177178, Abcam), anti-H3K4me1 (A2355, Abclonal), mouse monoclonal anti-FBP1 (DF7F25, Affinity) and anti-beta-actin (AF7018, Affinity) antibodies; secondary antibodies against mouse and rabbit IgG were purchased from Santa Cruz Biotechnology.

### Plasmids and transfection

The expression plasmids of miR-24-1 were built by inserting the fragments of pre-miR-24-1 (68 bp) and pri-miR-24-1 (708 bp) amplified from genomic DNA in HEK293T cells into the multiple cloning sites in the pSUPER-retro-GFP/Neo and pCDH-CMV-MCS-EF1-copGFP vectors. The miR-24-1 mutant plasmids were generated by using a One Step Cloning Kit (C114, Vazyme). The enhancer region (chr9:95084940-95087024) containing the miR-24-1 DNA locus was cloned into the luciferase reporter gene vector pGL3-Basic (Promega) to generate the reporter construct pGL3-enhancer. Target cells with strong GFP positivity were screened by flow cytometry (BD Biosciences). CRISPR/Cas9 vectors containing both GFP and puromycin resistance genes were obtained from Sangon. Lentivirus was produced by cotransfecting 293T cells with psPAX2, pMD2G and the pCDH-copGFP expression vector and harvested by filtration through a 0.45 μm filter (Millipore) after 72 hours of incubation. Stably transfected cells with strong GFP fluorescence were selected by flow cytometry (BD).

### Tissue samples

Renal carcinoma and adjacent normal tissue sections were obtained with informed consent under the approval by the Institutional Review Board of The Fourth Military Medical University. All the patients gave informed consent. Between May 2019 and October 2020, 42 patients pathologically diagnosed with RCC at Xijing Hospital were selected. Tissue sections were collected, immediately placed and stored in liquid nitrogen.

### RNA extraction and qRT-PCR

Total RNA extraction was gained by TRIzol reagent (Invitrogen, 15,596,018), purified, eluted in RNase-free water, and subsequently reverse transcribed into cDNA with a PrimeScript RT Reagent Kit with gDNA Eraser (Takara) under the instructions. A SYBR Green qRT-PCR master mix kit (TIANGEN) was utilized to perform qPCR according to the manufacturer’s procedures. All primer sequences are listed in **Table 1**. Ct values obtained from qPCR were used to calculate the relative expression level of all reported genes *via* the 2 ^−ΔΔCt^ method.

### Western blotting

Total protein from cells and tissues was extracted by using radioimmunoprecipitation assay (RIPA) buffer (TIANGEN). Protein concentrations were detected by a BCA Protein Assay Kit (TIANGEN). Protein lysates were loaded on a 4-12% gradient sodium dodecyl sulfate (SDS)-polyacrylamide gel (Life Technologies) and were then transferred onto polyvinylidene difluoride (PVDF) membranes (Millipore, Billerica). The immunoblots were probed with primary antibodies against FBP1 (1/2,000 dilution, Affinity) and beta-actin in 5% milk and were then reacted with horseradish peroxide (HRP)-conjugated donkey anti-rabbit or anti-mouse secondary antibodies (1/2,000 dilution; Amersham). Then immunoreactions were detected with an enhanced chemiluminescence (ECL) system.

### CRISPR/Cas9 system

CRISPR/Cas9 vectors (Sangon) targeting the enhancer region containing the miR-24-1 DNA locus of FBP1 were transfected into 786-O and ACHN cells to delete the targeted fragment. Dual guide RNAs (5’-TGTCGATTGGACCCGCCCTC-3’ and 5’-ACACACTGGCTCAGTTCAGC-3’) were designed from the Zhang laboratory’s public instructions (https://zlab.bio/guide-design-resources). The transfected cells were subjected to PCR to obtain the potentially deleted fragments. Then, the PCR products were sequenced by Sanger sequencing to determine the targeting efficiency of the constructed plasmids. Cells with effective deletion were separated through flow cytometry into 6 cm dishes and cultured in 5% CO2 at 37°C.

### ChIP and ChIP-qPCR

The stable cells were fixed with 1% formaldehyde for 15 min at room temperature. Subsequently, the cells were coated with 0.125 M glycine solution to quench the formaldehyde crosslinking reaction for 15 min. Cold PBS was used to wash the cell for twice. The cells were transferred into 15mL Corning tubes and resuspended in lysis buffer plus protease inhibitor cocktail (Roche) to obtain nuclear extracts. Nuclear lysis buffer was added to the extracted products for sonication. After sonication, the acceptable DNA fragments were incubated with specific antibodies against H3K4me1 and H3K27ac along with Protein A Dynabeads (Invitrogen) at 4°C overnight. Immunoprecipitated DNA fragments were washed sequentially with high- and low-salt wash buffers to wash the beads. After decrosslinking at 65°C for 6 hours, DNA fragments were purified with a DNA purification kit (Qiagen) to obtain ChIP template DNA. qPCR reactions were performed in a LightCycler 96 System (Roche). The sequences of the chromatin region-specific primers are listed as in **Table 1**.

**Table 1:** The primer sequences used for plasmids construction, RT-PCR and ChIP-qPCR assays.

Primer Names	Sequence (5’→3’)
pri-miR-24-1-F	GAAGATTCTAGAGCTAGCGAATTCGTCTGTCCACAGAAACATGCAC
pri-miR-24-1-R	GCAGATCCTTCGCGGCCGCGGATCCACACGCACCCACTCTAAC
pre-miR-24-1-F	ATCCGAGCTCGGTACCAAGCTTCTCCGGTGCCTACTGAGCT
pre-miR-24-1-R	AGATCGATCTCTCGAGGTCGACCTCCTGTTCCTGCTGAACTG
miR-24-1-3p-F	CGTCAGCTGTCCGAGTAGAGGtGGCTCAGTTCAGCA
miR-24-1-3p-R	TGTCAGGCAACCGTATTCACCcTGTTCC
FBP1-F	ACCCTGCCGTCACTGAGTA
FBP1-R	GCCCCATAAGGAGCTGAAT
GAPDH-F	ACCGTCAAGGCTGAGAAC
GAPDH-R	GCCTTCTCCATGGTGGTGA
GLUT1-F	GGTTGTGCCATACTCATGACC
GLUT1-R	CAGATAGGACATCCAGGGTAGC
LDHA-F	CCGTTACCTAATGGGGGAAA
LDHA-R	GCAACATTCATTCCACTCCA
miR-24-1-ChIP-F	CCGGTGCCTACTGAGCTGAT
miR-24-1-ChIP-R	TCGGGCACTTACAGACACGA

### Dual luciferase reporter assays

The constructed pGL3-enhancer or control plasmids were cotransfected with the miR-24-1 expression vector and Renilla luciferase reporter vector pRL-SV40 into HEK293T cells. Then, the cells were harvested and lysed in lysis buffer (Promega) after transfection for 48 hours and were concentrated to obtain the supernatants. The relative luciferase units (RLU) of the supernatants were determined by using a Dual Luciferase Reporter Assay System (Promega). Renilla luciferase was applied for normalization. The ratio of firefly/Renilla luciferase units was calculated to indicate the relative enhancer activity. All luciferase assays were repeated in triplicate.

### Glucose consumption and lactate production assays

Glucose (2 g) purchased from Sangon (A501991) was dissolved in 20 mL of glucose-free medium and prepared into a glucose mother solution. One milliliter of the glucose mother solution was added to 49 mL of medium to prepare 2000 mg/L glucose medium. miR-24-1 overexpressing and control 786 O and ACHN cells were seeded in 6-well plates (1×10^5^ cells/well) and cultured in DMEM. After 24 hours of culture, Glucose consumption and lactate production were measured with lactate assay kit and glucose test kit (Nanjing Jiancheng Bioengineering Institute) with the manufacturer’s protocol.

### Seahorse glycolysis stress test

miR-24-1 overexpressing and control 786 O and ACHN cells (1.5×10^4^ cells/well) were seeded into XFe24 cell culture microplates (Seahorse Bioscience) in DMEM. The cells were cultured overnight for 12 hours and were then cultured for 2 hours in the absence of glucose before Extracellular acidification rates (ECARs) were quantified using an XFe24 instrument (Seahorse Bioscience) under the protocol. After the Seahorse experiment, the BCA protein quantitation kit was used to normalize the data.

### Cell proliferation, colony formation, and transwell assays

Cells were placed in 96-well plates with 5,000 cells per well in DMEM and assessed daily (24, 48, 72, and 96 hours) with a Cell Counting Kit-8 (CCK-8, Dojindo). The proliferation ability was calculated by measuring the optical density (OD) (490 nm). Besides, for the colony formation assay, a total of 500 cells were plated in each well of 6-well plates. After incubation for 14 days, the formed colonies were stained with 0.25% crystal violet, imaged, and counted, and the number of colonies was reported. For the transwell migration assay, 1×10^4^ cells were transferred into each plastic insert of 24-well plates. In this two-chamber system, serum-free DMEM was added to the upper chamber, and 20% FBS-DMEM was added to the lower chamber. After 24 hours, the cells that traversed the membrane into the lower chamber were fixed with 100% methanol, stained with 0.1% crystal violet solution, imaged, and counted under a microscope. All assays were repeated in triplicate.

### Animals and *in vivo* experiments

A total of 18 male nude mice in six-week-old age were purchased from the Experimental Animal Center of Fourth Military Medical University and divided randomly into three groups. All the nude mice were maintained in the Experimental Animal Center of Fourth Military Medical University under the appropriate conditions: Temperature: 22 ± 1°C; Humidity: 50 ± 10%. miR-24-1 overexpression, miR-24-1 overexpression with enhancer deletion and control ACHN cells were injected into 6 male nude mice in the left hind limb (4×10^6^ cells per mouse), respectively. The tumor volume was measured every three days after 2 weeks of feeding. After another 4 weeks, tumor tissues were harvested for further analyses. The pathology of the tissues was confirmed by hematoxylin-eosin (HE) staining.

### HE staining, tissue immunohistochemistry and immunofluorescence

Human and mouse tissues were soaked in 10% neutral formalin for 72 hours. Then, paraffin embedding was carried out according to the routine procedure, and HE staining analysis was carried out after the samples were dehydrated.

Tissue sections were dewaxed and washed with distilled water. Tissue sections were placed in citric acid antigen repair buffer (pH=6.0) for antigen repair in a microwave. The sections were then washed with PBS for three times after natural cooling. Then, 3% hydrogen peroxide solution was added to block endogenous peroxidase activity in the dark at room temperature for 25 min. Next, 3% bovine serum albumin (BSA) was added to cover the tissues to block nonspecific binding for 30 min. After three washes in PBS, the primary antibody was added to incubate with the tissues overnight at 4°C. Next, the slides were incubated with the secondary antibody for 50 min. For immunohistochemistry, the slides were processed with DAB solution (Wuhan Servicebio Technology) and hematoxylin solution. For immunofluorescence, the slides were processed with DAPI solution (Wuhan Servicebio Technology) and a spontaneous fluorescence quenching reagent. Finally, the cells were photographed with a fluorescence microscope (Olympus BX53).

### The analysis of high throughput sequencing of human RCC samples from TCGA dataset

Tissue samples and their paired noncancerous matched tissues in current study for mRNA-seq were acquired from TCGA database. Differently expressed genes (DEGs) were identified with the R package DESeq2. Log2 (Fold change) > 1 and p < 0.05.

Available DNA methylation data (450k) of TSGs for RCC were downloaded from TCGA database. The difference between the mean methylation levels of paired samples >5% was considered to be of significance followed by Wilcoxon Rank Sum Test.

### Statistical analysis

All results are presented as the mean standard deviation (s.d.) of triplicate experiments unless otherwise noted. Data analysis was performed with a two-tailed Student’s t-test. **** means p < 0.0001, *** means p < 0.001, ** means p < 0.01 and * means p < 0.05. p < 0.05 was considered significant. GraphPad Prism (Version 7.0, GraphPad Software, Inc.) was utilized to perform statistical analysis.

## Results

### *FBP1* is downregulated in RCC and related to poor prognosis

**Figure 1:**
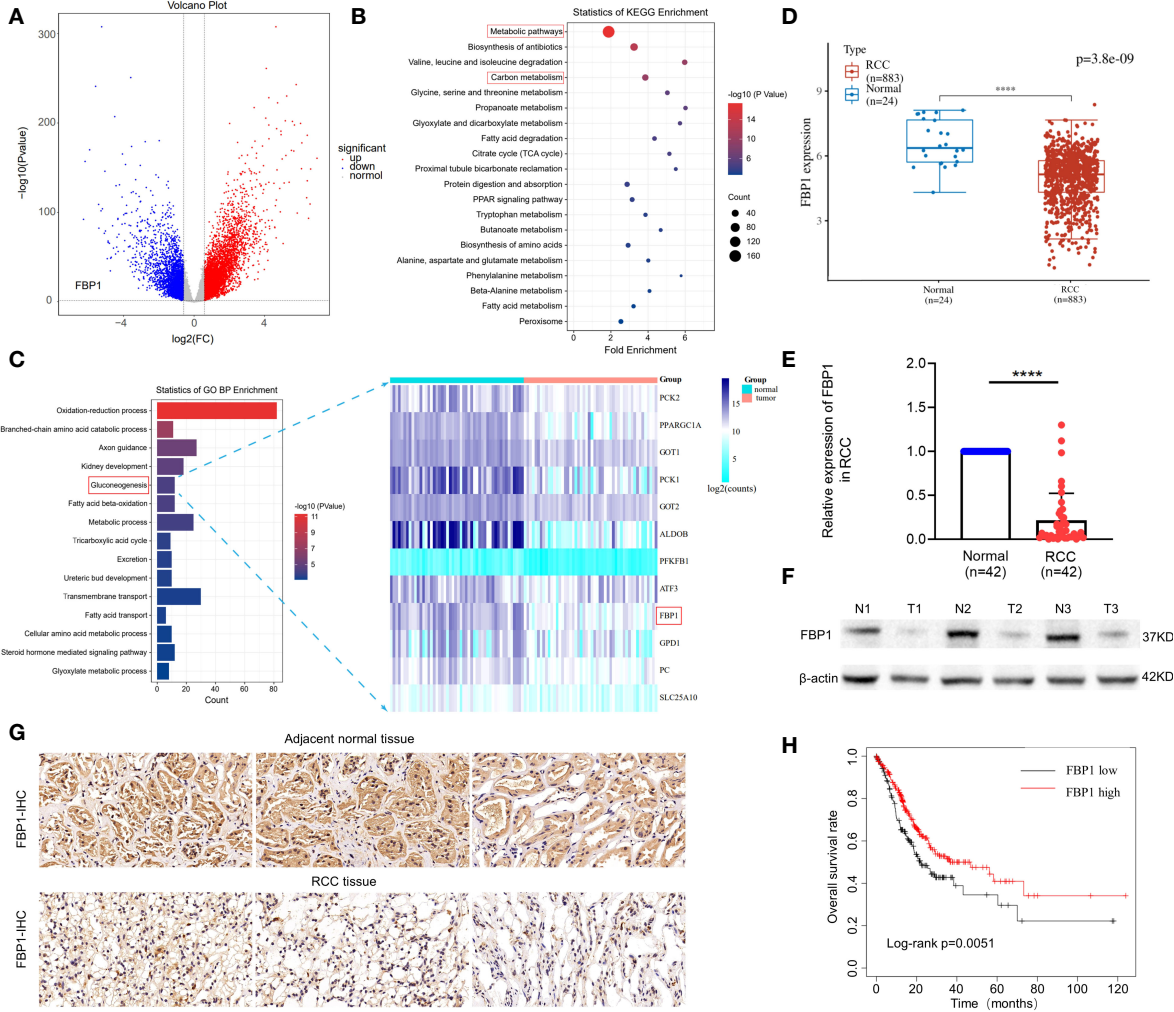
*FBP1* is reduced in RCC and related to poor survival outcome. **(A)** Volcano Plot of differentially expressed mRNAs associated with glucose metabolism of Warburg effect in RCC and adjacent normal tissues. Blue represents downregulated genes, and red represents upregulated genes. p < 0.05. **(B, C)** KEGG pathway and gene oncology (GO) profiling for the downregulated genes in RCC. **(D)** The mRNA expression levels of FBP1 in RCC and adjacent normal renal tissues from TCGA database by bioinformatic analysis. **(E)** The mRNA expression levels of FBP1 in RCC and adjacent normal renal tissues detected by qPCR. **(F)** The protein level of FBP1 in 3 randomly selected paired RCC and adjacent normal renal tissues by western blot. b-actin was used as input control. **(G)** Representative pictures of immunohistochemical (IHC) staining of 3 randomly selected paired RCC tissue and adjacent normal renal tissues. **(H)** Kaplan–Meier plots of overall survival in RCC patients with low expression of *FBP1* (n=166) compared to high expression of FBP1 (n=178) stratified by the expression levels of *FBP1*. The cutoff was confirmed as the threshold with the best performance. Log-rank test p value is shown. Results are shown as mean ± S.D., ****p < 0.0001.

To investigate the expression patterns of *FBP1* in kidney cancer, we analyzed the mRNA-seq datasets of 611 RCC and normal renal control samples from the TCGA database. As expected, *FBP1* was downregulated in RCC, as shown in **Figure 1A** (blue panel, p < 0.05, log2FoldChange > 1). Interestingly, we discovered that genes encoding key factors in glucose metabolism involved in the Warburg effect, such as *HK2*, *HK3* and *LDHA*, were significantly upregulated in RCC compared to adjacent normal tissues (red panel, **Figure 1A**). This result is consistent with their positive role on Warburg effect in other cancer types (21–23). Next, KEGG pathway and gene oncology (GO) profiling for the downregulated genes in RCC revealed they are mainly related to metabolic pathways and gluconeogenesis in **Figures 1B, C**, respectively. It is noteworthy that 12 gluconeogenesis-associated genes like *PCK1* and *PCK2* are also lowly expressed other than *FBP1* in RCC as seen in **Figure 1C** (right panel), further confirming the expression of gluconeogenesis-associated genes is aberrant in RCC.

Then, to determine the mRNA expression level of *FBP1* in tissue samples, 42 paired RCC and matched adjacent tissues were utilized for qPCR detection. Consistent with our expectation, *FBP1* was obviously downregulated in RCC compared to normal tissues, as shown in **Figures 1D**, **E**. In addition, we examined the differences in protein levels between 3 paired RCC and adjacent normal tissues that were randomly selected from the 42 paired tissue samples. The FBP1 protein was expressed at significantly lower levels in the RCC tissues than in the adjacent normal ones (**Figure 1F**). Furthermore, immunohistochemical staining assays revealed that FBP1 was obviously expressed at lower levels in RCC tissues than in normal tissues (**Figure 1G**). Since *FBP1* was confirmed to be downregulated in RCC, we assume that it may act as a suppressor of RCC. Therefore, Kaplan–Meier plots of overall survival were analyzed to confirm the effects of *FBP1* expression on patient survival outcomes (**Figure 1H**). In the TCGA dataset, patients with RCC with lower *FBP1* expression exhibited worse survival outcomes (**Figure 1G**), suggesting that *FBP1* indeed exhibits a tumor-suppressive effect.

### Tumor suppressor gene FBP1 is upregulated by miR-24-1 in RCC cells

**Figure 2:**
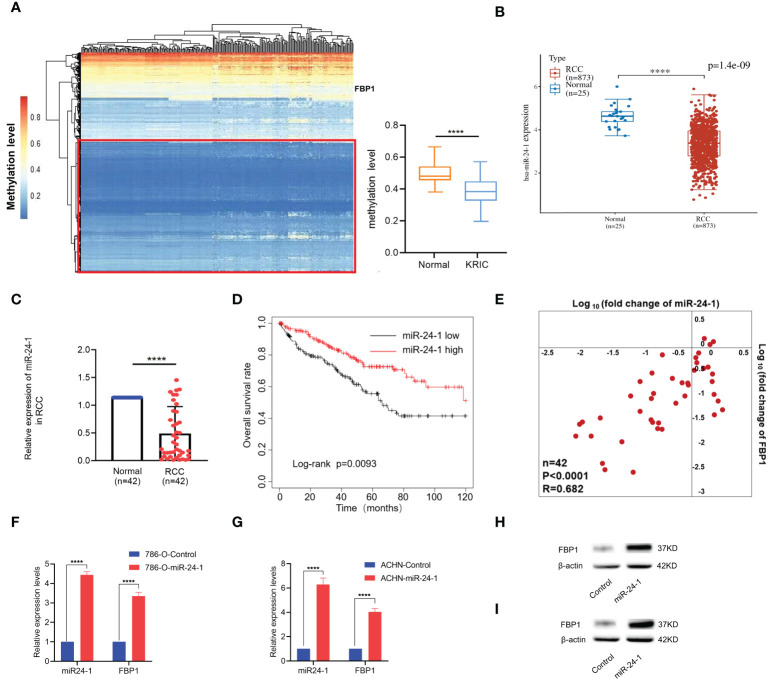
Tumor suppressor gene *FBP1* can be activated by miR-24-1 in RCC cells. **(A)** Left panel: DNA methylation profiling for downregulated tumor suppressor genes in RCC, reporting the depletion of *FBP1* in RCC was not caused by hypermethylation in promoter; Right panel: the methylation level of *FBP1* promoter between normal and KIRC ones. **(B)** The expression levels of miR-24-1 in RCC and adjacent normal renal tissues from TCGA database by bioinformatic analysis. **(C)** The expression levels of miR-24-1 in RCC and adjacent normal renal tissues detected by qPCR. **(D)** Kaplan–Meier survival analysis showed miR-24-1 is significantly associated with poor overall survival in RCC patients with low expression of miR-24-1 (n=166) compared to high expression of miR-24-1 (n=265). **(E)** Both miR-24-1 and *FBP1* are downregulated in RCC compared to normal ones. Besides, the expression levels of miR-24-1 and *FBP1* in RCC tissues exhibit a significantly positive correlation. Correlation coefficient = 0.682. **(F, G)** The activation of *FBP1* by miR-24-1 was assessed by qPCR in 786-O **(F)** and ACHN **(G)** cells after transfecting miR-24-1 expression vectors. **(H, I)** The protein levels of FBP1 in 786-O **(H)** and ACHN **(I)** cells were increased after transfecting miR-24-1 confirmed by western blot. Results are shown as mean ± S.D., ****p < 0.0001.

Epigenetic regulations like DNA hypermethylation exert significant roles on inactivation of tumor suppressor genes without altering DNA sequence (24). Loss of function of tumor suppressor genes may eventually contribute to tumorigenesis (25). Therefore, we firstly carry on DNA methylation profiling for 491 downregulated tumor suppressor genes in kidney renal clear cell carcinoma (KIRC) from TSGene database (https://bioinfo.uth.edu/TSGene/) (26). Available DNA methylation data with KIRC were downloaded from TCGA database. Interestingly, the results showed 61% tumor suppressor genes exhibited hypomethylation but with no methylation difference as seen in left panel of **Figure 2A** (red box). Particularly, *FBP1* showed DNA hypermethylation status in promoter regions in both KIRC and normal ones, which is even relatively higher in normal than that in KIRC (right panel of **Figure 2A**), implying that *FBP1* depletion in renal cell carcinoma is not caused by DNA hypermethylation in promoter.

Based on our previous work, NamiRNA plays positive regulatory function on tumor suppressor genes through enhancer (16) and in particular, miR-24-1 can activate its neighboring gene *FBP1* in 293T cells (13). So, we put our attention to the regulatory function of miR-24-1 on *FBP1* to investigate its potential role in RCC. Subsequently, we examined the mRNA expression level of miR-24-1 in 873 RCC patients and 25 normal controls from TCGA by bioinformatic analysis. Interestingly, miR-24-1 expressed in lower level in RCC tissues than that in adjacent normal tissues (**Figure 2B**). Next, we detected the expression of miR-24-1 by qPCR. The results confirmed that miR-24-1 was significantly downregulated in the 42 RCC tissues compared to the adjacent normal tissues (**Figure 2C**). In addition, Kaplan–Meier analysis of overall survival rate showed that patients with low miR-24-1 expression had obviously poor survival outcomes in the TCGA datasets (**Figure 2D**). Finally, we calculated the correlation coefficient of the expression levels for *FBP1* and miR-24-1 in tumor tissues. The result showed their expressions are significantly positively correlated (R=0.682), implying *FBP1* may be positively regulated by miR-24-1 (**Figure 2E**). Taken together, these findings indicated that both *FBP1* and miR-24-1 are downregulated in RCC and that their downregulation can predict poor survival in RCC.

As *FBP1* can be upregulated by miR-24-1 in HEK293T cells (13), we examined whether miR-24-1 exerts an activating effect on *FBP1* in the RCC cell lines 786-O and ACHN. Lentiviruses overexpressing miR-24-1 (GFP+) and empty control vector (GFP+) were transfected into 786-O and ACHN cells to obtain stable cell lines. Accordingly, the expression levels of miR-24-1 in 786-O and ACHN cells were increased by 4-fold and 6-fold, respectively; moreover, *FBP1* expression was increased by 3.2-fold and 4-fold, respectively, after miR-24-1 overexpression (**Figures 2F, G**). The protein expression levels of FBP1 were confirmed in 786-O and ACHN cells by western blot analysis and were consistent with the above result (**Figures 2H, I**). These results showed *FBP1* can be upregulated by miR-24-1 in RCC cells.

### MiR-24-1 suppresses the proliferation and migration of RCC cells by activating FBP1

**Figure 3:**
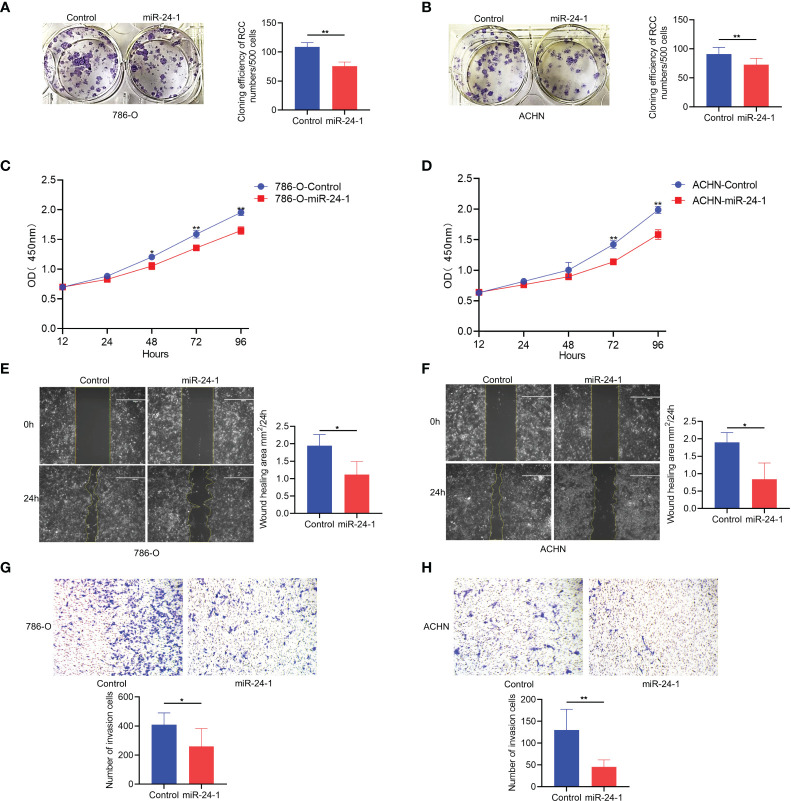
*FBP1* can be activated by miR-24-1 and inhibit the proliferation and migration of RCC cells. **(A, B)** The colony formation ability of 786-O **(A)** and ACHN **(B)** cells was inhibited after overexpressing miR-24-1 in colony formation assay. **(C, D)** The proliferation ability of 786-O **(C)** and ACHN **(D)** cells was blocked after overexpressing miR-24-1 in CCK8 assay. **(E, F)** The migrating abilities of 786-O **(E)** and ACHN **(F)** cells were inhibited after overexpressing miR-24-1 in would healing assay. **(G, H)** The migrating and invasive abilities of 786-O **(G)** and ACHN **(H)** cells were repressed after overexpressing miR-24-1 in transwell assay. Results are shown as mean ± S.D. of triplicated experiments, **p < 0.01, *p< 0.05.

To investigate how *FBP1* activation by miR-24-1 affects the proliferation of 786-O and ACHN cells, colony formation assays were performed by utilizing the above stable cell lines. The observations showed that transfection of miR-24-1 reduced the number of colonies formed by both 786-O and ACHN cells after incubation for 14 days (**Figures 3A, B**). In addition, a CCK-8 assay was carried out to characterize cell viability at different timing (12, 24, 48, 72 and 96 hours, respectively). 786-O cells with overexpressing miR-24-1 showed a higher growth rate than the corresponding control group at 48, 72, and 96 hours, and ACHN cells overexpressing miR-24-1 showed a higher growth rate than the corresponding control cells at 72 and 96 hours (**Figures 3C, D**). These results indicate *FBP1* activation by miR-24-1 can repress the proliferation and growth of RCC cells. In turn, transfecting miR-24-1 inhibitor can suppress *FBP1* expression (**Supplementary Figures 1A, B**) and promotes the proliferation and growth of RCC cells (**Supplementary Figures 1C, D**).

In addition to validating its effects on the proliferation and growth of RCC cells, we also assessed whether miR-24-1 overexpression affects the migration ability of them. The wound healing assay revealed that the wound areas of 786-O and ACHN cells overexpressing miR-24-1 closed significantly slower than those of the corresponding control cells after incubation for 24 hours (**Figures 3E, F**). Furthermore, the numbers of 786-O and ACHN cells with miR-24-1 overexpression that migrated and traversed the membrane into the lower chamber were obviously lower than those of the corresponding control cells in the transwell assay (**Figures 3G, H**), indicating that *FBP1* activation by miR-24-1 overexpression can block the migrating ability of RCC cells. Overall, these results suggest FBP1 activated by miR-24-1 plays an inhibitory role in the proliferation and migration of RCC cells.

### Transcriptional activation of FBP1 by miR-24-1 is mechanistically triggered by an enhancer

**Figure 4:**
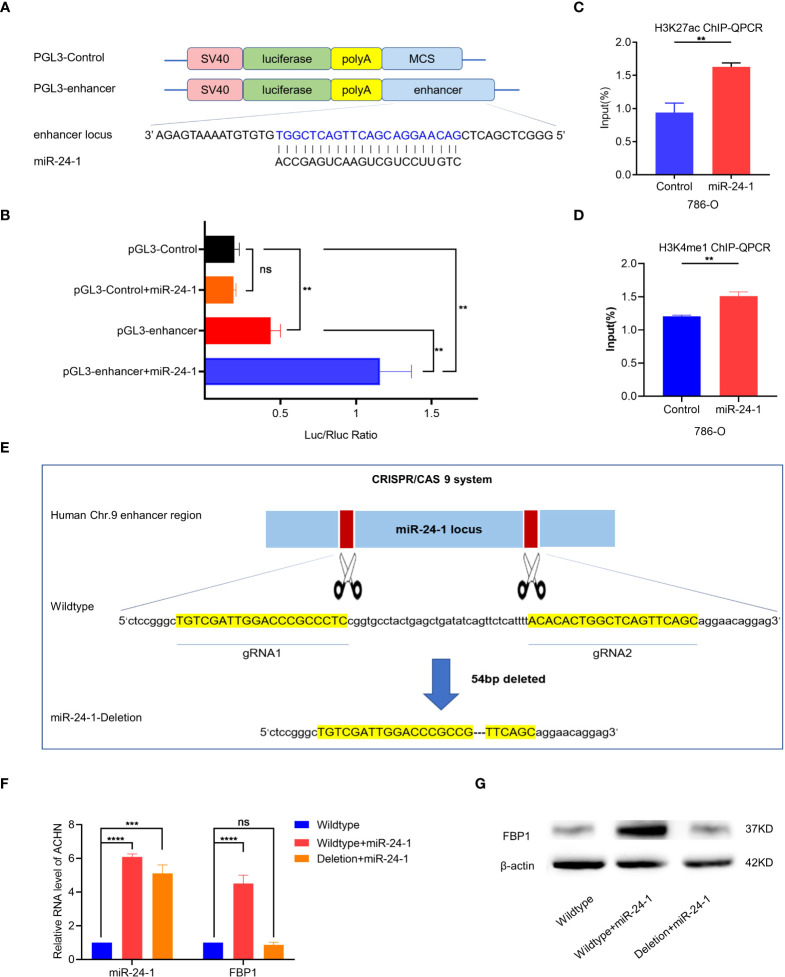
MiR-24-1 mechanistically activates *FBP1* through enhancer. **(A)** The schematic diagram of pGL3-enhancer vector construction in dual luciferase reporter gene assay. The enhancer sequence containing miR-24-1 DNA locus located on 60 kb upstream from *FBP1* was inserted into pGL3 vector to construct pGL3-enhancer vector. **(B)** The enhancer sequence containing miR-24-1 DNA locus can increase the activity of reporter gene. Co-transfection of miR-24-1 expression vector and pGL3-enhancer vector induced an increase of enhancer activity in dual luciferase reporter gene assay. **(C, D)** More enrichment of H3K27ac **(C)** and H3K4me1 **(D)** on miR-24-1 locus was observed by ChIP-qPCR after overexpressing miR-24-1. **(E, F)** The schematic diagram of CRISPR/Cas9 system. A deletion of 54bp in enhancer sequence containing miR-24-1 **(E)** was confirmed by Sanger sequencing **(F)**. **(G)** The mRNA expression levels of *FBP1* were detected by qPCR. *FBP1* was increased when overexpressing miR-24-1, yet failed to be activated when enhancer was deleted. **(G)** Similarly, the protein expression levels of FBP1 were detected by western blot. Results are shown as mean ± S.D., ****p < 0.0001, ***p < 0.001, **p < 0.01, ns means not significant.

Enhancers overlapping with miRNA loci crosstalk with the corresponding miRNAs to increase the expression of miRNAs crucial for cell identity (15). Hence, we hypothesized that miR-24-1 increases enhancer activity and, thus, this enhancer activates transcriptional expression of *FBP1*. To test our hypothesis, we first inserted the enhancer region containing the miR-24-1 DNA locus into the pGL3 vector to construct the pGL3 enhancer and detected enhancer activity by a dual luciferase reporter assay (**Figure 4A**). As shown in **Figure 4B**, the enhancer sequence indeed exhibited enhancer activity after transfection with the pGL3-enhancer vector compared to the control (**Figure 4B**). In addition, when the pGL3-enhancer vector was co-transfected with the miR-24-1 expression vector, the reporter activity of was obviously increased compared to that in cells transfected with only the pGL3-enhancer vector (**Figure 4B**), suggesting that miR-24-1 can boost enhancer activity to activate the reporter.

Furthermore, we evaluated the alterations in H3K27ac and H3K4me1 enrichment at the miR-24-1 locus by ChIP-qPCR, because H3K27ac and H3K4me1 are typical enhancer markers. A greater enrichment of H3K27ac and H3K4me1 at miR-24-1 loci was observed in miR-24-1-overexpressing cells (**Figures 4C, D**). Taken together, these results demonstrated that miR-24-1 can increase enhancer activity to activate the expression of the target gene FBP1. Conversely, we examined whether deletion of the enhancer region (54 bp) by CRISPR/Cas9 gene editing exerts a negative effect on the regulation of *FBP1* by miR-24-1 (**Figures 4E, F**). As expected, in wild-type ACHN cells, miR-24-1 overexpression increased the expression of *FBP1*; however, after deletion of the enhancer, overexpression of miR-24-1 no longer activated *FBP1* in ACHN cells compared to wild-type ACHN cells, as determined by qPCR (**Figure 4F**). Consistent with the alterations in mRNA expression, FBP1 protein expression was not activated, as determined by western blot analysis (**Figure 4G**).

### *FBP1* reactivation disrupts Warburg effect to suppress kidney cancer

**Figure 5:**
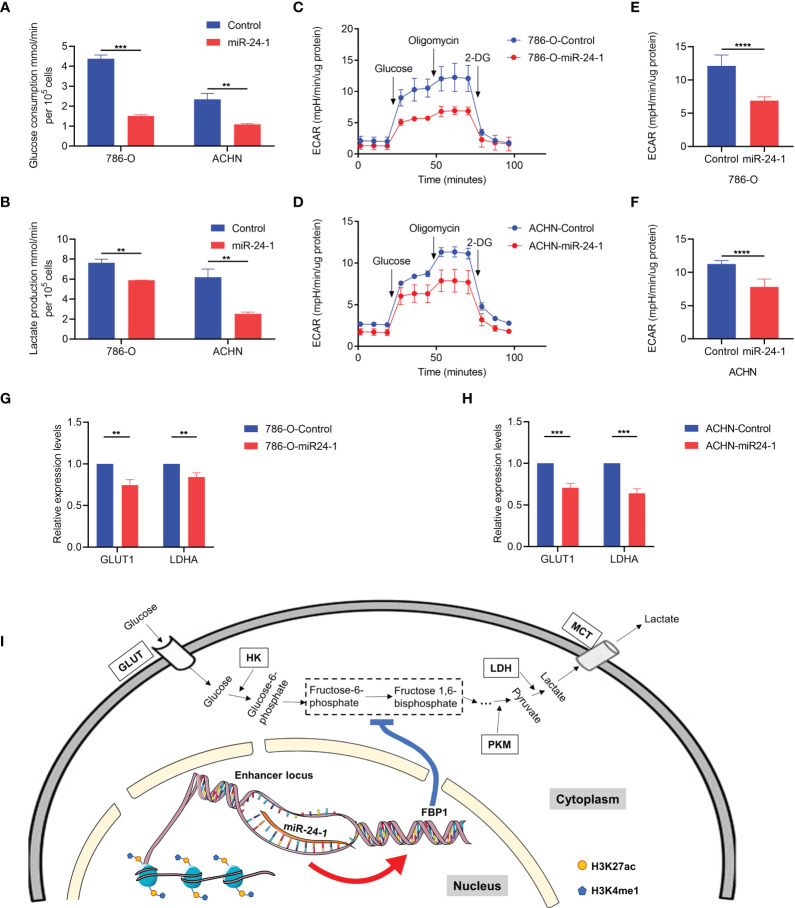
*FBP1* suppresses Warburg effect in RCC cells. **(A)** Quantification of glucose consumption in miR-24-1-overexpressing 786-O and ACHN cells. **(B)** Lactate production in 786-O and ACHN cells transfected with miR-24-1 was measured by lactate assay. **(C, D)** Extracellular acidification rate (ECAR) of 786-O **(C)** and ACHN **(D)** cells was detected after miR-24-1 overexpression with glucose starved for two hours and subsequently treated with 2 g/L D-glucose, 1 μM oligomycin, and 100 mM 2-Deoxyglucose (2-DG). **(E, F)** ECAR represents maximum glycolytic capacity after oligomycin treatment of 786-O **(E)** and ACHN **(F)** cells. **(G, H)** qPCR analysis of glycolytic related genes in vector control or miR-24-1-overexpressing 786-O **(G)** and ACHN **(H)** cells. **(I)** Schematic diagram of *FBP1* being activated by miR-24-1 to disturb Warburg effect in RCC cells. The enriched modifications of H3K4me1 and H3K27ac represent active enhancer markers. Results are shown as mean ± S.D. of triplicated experiments, ****p < 0.0001, ***p < 0.001, **p < 0.01.

FBP1, a rate-limiting enzyme in gluconeogenesis, catalyzes the hydrolysis of fructose 1,6-bisphosphate to fructose 6-phosphate, playing a critical role in the energy metabolism of cancer cells. To investigate whether *FBP1* blocks RCC progression through the Warburg effect, the levels of glucose consumption and lactate production were measured in RCC cell lines. It showed that miR-24-1 inhibits aerobic glycolysis by activating *FBP1*, as indicated by the decreases in glucose consumption and lactate production in 786-O and ACHN cells (**Figures 5A, B**). To further evaluate the impact of miR-24-1 and *FBP1* on aerobic glycolysis in RCC cells, the ECAR was measured with an XFe24 extracellular flux analyzer (Seahorse). The glycolytic ECAR was measured immediately following the addition of glucose after the cells were glucose starved for approximately 2 hours. The maximum glycolytic capacity is equal to the ECAR after oligomycin treatment. Transfection of miR-24-1 can reduce the ECAR (**Figures 5C, D**) and the maximum glycolytic capacity (**Figures 5E, F**) of 786-O and ACHN cells. Moreover, we detected the mRNA levels of the glycolysis-related genes *LDHA* and glucose transporter 1 gene *GLUT1* which are two key factors in Warburg Effect after transfecting miR-24-1 in 786-O and ACHN cells. The results showed activation of *FBP1* by miR-24-1 further declined the expression of *LDHA and GLUT1* (**Figures 5G, H**). Collectively, these findings indicated that overexpression of miR-24-1 activates *FBP1* transcription by targeting active enhancers in the nucleus, and reactivated *FBP1* then inhibits Warburg effect in cancer cells by slowing aerobic glycolysis, which finally blocks RCC progression (**Figure 5I**).

### The *FBP1*/miR-24-1/enhancer axis inhibits kidney tumor growth in mice

Since *FBP1* can be activated by miR-24-1 expression induced by enhancer elements, we further studied the function of the *FBP1*/miR-24-1/enhancer axis in tumor growth *via* xenograft experiments *in vivo*. First, ACHN cells stably expressing miR-24-1 with deletion of the enhancer region and the corresponding control cells were orthotopically injected into the flanks of male nude mice to test the function of miR-24-1 in tumor growth (4×10^6^ cells/mouse, three groups of mice, n=6 mice/group). Notably, successful overexpression of miR-24-1 was demonstrated in these stable cells to increase *FBP1* expression (**Figures 2F, G**) or fail to increase FBP1 expression due to deletion of the enhancer region (**Figure 4G**). The growth rate was calculated and reported as the tumor volume during the treatment process.

**Figure 6:**
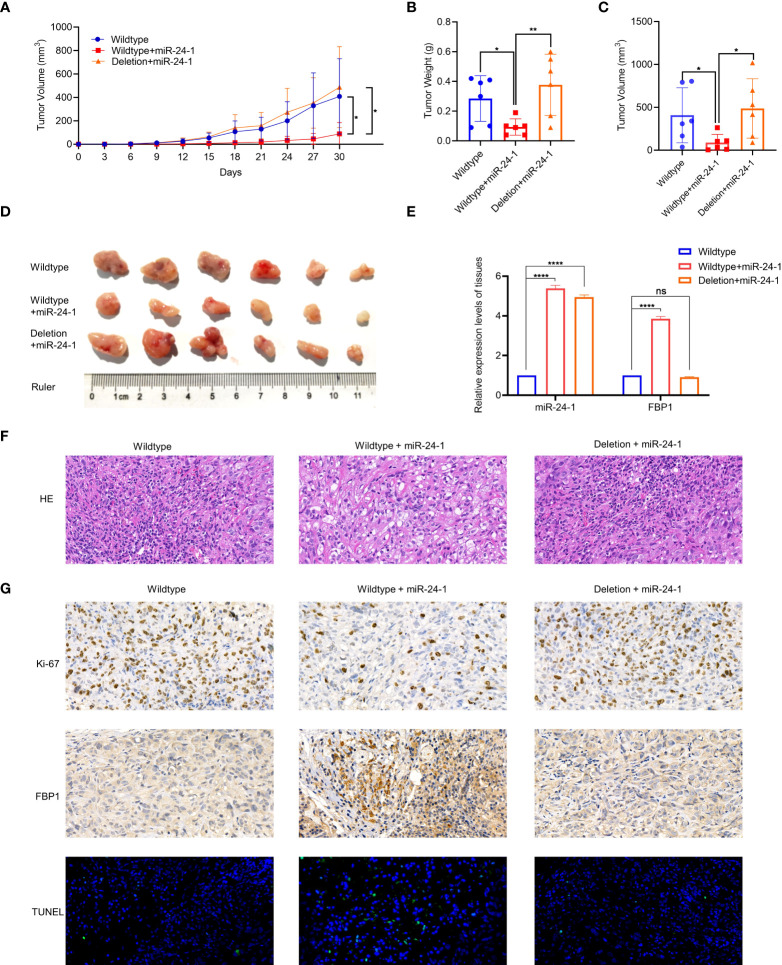
MiR-24-1 activates *FBP1 via* enhancer to suppress tumor growth *in vivo*. **(A)** Measuring tumor sizes every 3 days during feeding period. The day when tumors were initially formed marked as day 0. Mice were sacrificed on day 30. **(B, C)** The weight and size of tumors were calculated. **(D)** The dissected tumors from sacrificed mice were photographed. **(E)** qPCR assay was performed to detect the expression levels of miR-24-1 and *FBP1* in the tumor tissues among three groups. **(F)** Representative images of HE staining of tumors. HE staining confirmed that the tumors in each group were indeed RCCs (magnification, 500×). **(G)** Representative images of immunohistochemistry of tumors. The expression of Ki-67 was obviously less in the tumors from overexpressing miR-24-1 group than those from control group and enhancer-deleted group (top). The expression of *FBP1* was increased by overexpressing miR-24-1, but not when enhancer was deleted (middle). The number of TUNEL-positive cells (green fluorescent signal) in the overexpressing miR-24-1 group was significantly increased, indicating an increase of the apoptosis of tumor cells in this group (bottom) (magnification, 500×). Results are shown as mean ± S.D., **p < 0.01, *p < 0.05, ****p < 0.0001, ns means not significant.

As observed in **Figure 6A**, the growth of tumors derived from miR-24-1-overexpressing ACHN cells was significantly inhibited compared to that of tumors derived from empty vector-transfected cells or cells with enhancer deletion. After 6 weeks, the mice were sacrificed to obtain tumors for further studies. The tumors from the miR-24-1 overexpression group were smaller and weighed less than those from the empty vector-transfected and enhancer deletion groups (**Figures 6B–D**), suggesting that miR-24-1 overexpression activated *FBP1* expression to block tumor growth in a manner dependent on enhancer integrity. Moreover, we performed qPCR assay to figure out the expression levels of miR-24-1 in the tumor tissues among three groups. As seen in **Figure 6E**, miR-24-1 was significantly upregulated in both the overexpressing miR-24-1 group and enhancer-deleted group after transfecting miR-24-1 lentivirus compared to control group, however, *FBP1* was only activated in the over-expressing miR-24-1 group but not in enhancer-deleted group even miR-24-1 was overexpressed. HE staining confirmed that the tumors in each group were indeed RCCs (**Figure 6F**); IHC staining revealed that there were fewer Ki-67-positive cells in the miR-24-1 overexpression group than in the empty vector-transfected and enhancer deletion groups. In turn, TUNEL-positive cells were significantly more abundant in the miR-24-1 overexpression group than in the other two groups (**Figure 6G**), indicating that miR-24-1 overexpression inhibits tumor growth and increases the number of apoptotic cells. In addition, the protein level of FBP1 was elevated obviously in miR-24-1 overexpression group but not in the enhancer deletion group as seen in **Figure 6G**. Collectively, these results indicate that *FBP1* can be activated by miR-24-1 and inhibit tumor growth in a manner dependent on enhancer integrity (**Figure 6G**).

## Discussion

Although a myriad of RCC investigations have already been conducted, challenges remain in understanding its underlying mechanism (27). Recent studies have shown that *FBP1* is a critical player in the malignancies (28–31). Glycogen Branching Enzyme 1 (GBE1)-mediated *FBP1* suppression *via* promoter methylation contributes to tumor progression in lung adenocarcinoma (LUAD) (32). Low expression of *FBP1* is directly related to a poor overall survival rate based on JavaScript:; a comprehensive profiling of TCGA dataset, indicating that *FBP1* may be considered a potential prognostic biomarker in RCC as a tumor suppressor (32). Importantly, as a tumor suppressor, *FBP1* is generally depleted in ccRCC (11); thus, upregulating the expression of *FBP1* is a potential therapeutic strategy for cancer. In our study, we found that *FBP1* is depleted in RCC and can be activated by miR-24-1, which implies a potential treatment strategy for RCC.

MiRNAs are tumor hallmarks that are only 21-23 *nt* in length, and play critical roles in immunity (33), metabolism (34), cell proliferation and differentiation (35), and tumor progression (36). Since the discovery of these short RNA molecules in *Caenorhabditis elegans (* 37) in 1990s, miRNAs have been recognized to negatively regulate gene expression by degrading or repressing their target mRNAs (38). However, recent studies have also shown that miRNAs are as well involved in gene activation (13, 39, 40). For example, enhancer-overlapped miRNAs can activate target genes through chromatin remodeling at enhancer regions (13). Our work demonstrated that overexpression of miR-24-1 can activate *FBP1* to block RCC proliferation and metastasis (**Figures 2F, G**), shedding a light on the unconventional role of miRNAs in malignancies. However, there is still a paucity of datasets about the crosstalk between miRNAs and enhancers.

Enhancers are *cis*-acting DNA sequences that can function as transcription factor binding platforms and increase gene transcription levels independent of their orientation and location (41, 42). We verified miRNAs overlapped within enhancer region are capable of activating target genes *via* binding to enhancers in a manner dependent on the enhancer integrity (**Figures 4F, G**). In addition, our work showed that deletion of miR-24-1 reduces enhancer activity in RCC, nevertheless, enhancer inactivation leads to low expression of *FBP1*, which in turn promoted the Warburg effect. Although Warburg effect phenotype is well established, its role in cancer metabolism progression is still incompletely defined. In our study, we found that activation of the tumor suppressor *FBP1* by miR-24-1 can inhibit Warburg effect in RCC, suggesting that blockade of metabolic processes such as Warburg effect through miR-24-1 constitutes a good therapeutic strategy for RCC.

In conclusion, the current study revealed that low expression of enhancer-associated miR-24-1 can contributes to inactivation of *FBP1*, and that *FBP1* depletion further facilitates Warburg effect, which eventually promotes RCC development. Taken together, our findings provide an alternative mechanism for the low expression of *FBP1* in RCC and a potential therapeutic strategy for RCC treatment.

## Data availability statement

The original contributions presented in the study are included in the article/**supplementary material**. Further inquiries can be directed to the corresponding authors.

## Ethics Statement

The studies involving human participants were reviewed and approved by ethics committee of Fourth Military Medical University. The patients/participants provided their written informed consent to participate in this study. The animal study was reviewed and approved by Ethics Committee of Fourth Military Medical University.

## Author contributions

DJ and YL performed the research and wrote the manuscript. WY and YW provided experimental conditions. JLY contributed to the financial support and administrative support. GH, WZ, and GZ analyzed the data. XD, DW, FY, LZ, and JRY modified the manuscript. DL, YZ, FW, and PM provided the clinical samples. All authors contributed to the article and approved the submitted version.

## Funding

Our work was supported by the Xijing Hospital subject booster plan translational medicine research projects (grant number XJZT13Z05), the Military medical innovation project (grant number 16CXZ023) and the National Natural Science Foundation of China (grant number 81672535) and the Shaanxi Provincial Key Research and Development Program (grant number 2021SF-053).

## Acknowledgments

The authors sincerely appreciate Baolong Zhang, Pengxu, Zhicong Yang, Mengxing Liu, Xiaoguang Ren, Ying Tong and Daoping Ru from Fudan University for their critical comments in manuscript preparation. We also thank laboratory staff Yan Zhao and Guo Chen from the State Key Laboratory of Cancer Biology of the Fourth Military Medical University for their hard work in Animals experiments.

## Conflict of Interest

The authors declare that the research was conducted in the absence of any commercial or financial relationships that could be construed as a potential conflict of interest.

## Publisher’s note

All claims expressed in this article are solely those of the authors and do not necessarily represent those of their affiliated organizations, or those of the publisher, the editors and the reviewers. Any product that may be evaluated in this article, or claim that may be made by its manufacturer, is not guaranteed or endorsed by the publisher.

## Supplementary material

Supplementary Figure 1 miR-24-1 inhibitor declines the expression of *FBP1* and promotes the proliferation and migration of RCC cells. **(a, b)** qPCR assay was performed to confirm that the expression of miR-24-1 was successfully declined after transfecting miR-24-1 inhibitor, and *FBP1* was accordingly downregulated in ACHN (a) and 786-O **(b)** cells. **(c, d)** The proliferation ability of 786-O (c) and ACHN (d) cells was promoted after transfecting miR-24-1 inhibitor in CCK8 assay. Results are shown as mean ± S.D. of triplicated experiments, ****p < 0.0001, **p < 0.01, *p< 0.05.
