# Supplementary figures and images for: FBP1 /miR-24-1/enhancer axis activation blocks renal cell carcinoma progression via Warburg effect
Source: Front Oncol. 2022 Aug 1;12:928373. doi: 10.3389/fonc.2022.928373 (PMC9376222; doi:10.3389/fonc.2022.928373)

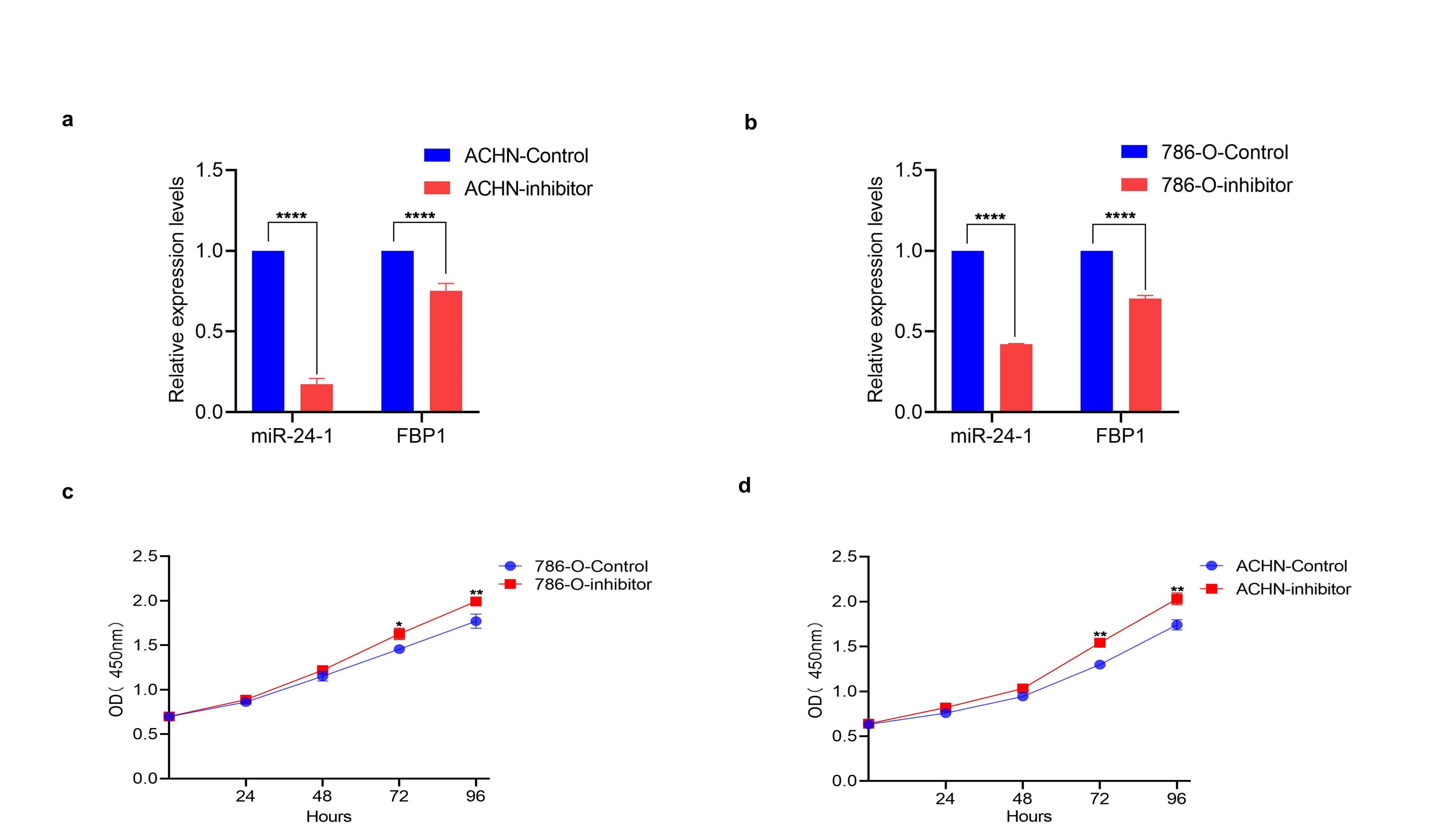

Supplement: Supplementary Figure 1 — miR-24-1 inhibitor declines the expression of FBP1 and promotes the proliferation and migration of RCC cells. (a, b) qPCR assay was performed to confirm that the expression of miR-24-1 was successfully declined after transfecting miR-24-1 inhibitor, and FBP1 was accordingly downregulated in ACHN (a) and 786-O (b) cells. (c, d) The proliferation ability of 786-O (c) and ACHN (d) cells was promoted after transfecting miR-24-1 inhibitor in CCK8 assay. Results are shown as mean ± S.D. of triplicated experiments, ****p < 0.0001, **p < 0.01, *p< 0.05. [file Image_1.jpeg]
